# Field surveys of egg mortality and indigenous egg parasitoids of the brown marmorated stink bug, *Halyomorpha halys*, in ornamental nurseries in the mid-Atlantic region of the USA

**DOI:** 10.1007/s10340-017-0890-8

**Published:** 2017-06-05

**Authors:** Ashley L. Jones, David E. Jennings, Cerruti R. R. Hooks, Paula M. Shrewsbury

**Affiliations:** Department of Entomology, University of Maryland, 4112 Plant Sciences Building, College Park, MD 20742 USA

**Keywords:** *Anastatus reduvii*, Native parasitoids, Biological control, Parasitism, Predation

## Abstract

The brown marmorated stink bug, *Halyomorpha halys* (Stål), is an invasive species native to regions of China, Japan, Korea, and Taiwan. In its native and introduced range, *H. halys* is considered a pest of tree fruits, vegetables, legumes, and ornamental trees. The highly polyphagous nature of this insect as well as its vast dispersal capabilities, require an integrated approach to management. Here we focus on the potential impact of indigenous natural enemies on *H. halys* in woody ornamental nurseries in Maryland, USA. We sampled naturally field laid *H. halys* egg masses for mortality and parasitism rates in 2012 and 2013. Overall, egg mortality averaged 54% for both years, and increased within season and between years. The largest source of mortality was from egg parasitoids with mean parasitism rates of 30.73 and 39.63% in 2012 and 2013, respectively. Mortality from predation was much lower and averaged 4.61% by chewing and 2.53% by sucking predators. We found seven species of Hymenopteran egg parasitoids attacking *H. halys* eggs, with *Anastatus reduvii* being the dominant species comprising 61.17 and 79.12% of all parasitoids in 2012 and 2013, respectively. The observed increase in parasitism over time and female biased sex ratio of parasitoids suggests that native parasitoids may be responding to the novel host, *H. halys*. Consequently, the use of these native eupelmid egg parasitoids in augmentative or conservation biological control may be a viable *H*. *halys* management strategy in ornamental nurseries.

**Electronic supplementary material:**

The online version of this article (doi:10.1007/s10340-017-0890-8) contains supplementary material, which is available to authorized users.

## Key message

- *Halyomorpha halys* is abundant in ornamental plant nurseries. Over 2 years, we found *H*. *halys* egg mortality averaging 54.1%, of which hymenopteran parasitoids (7 species total) were the primary mortality source (35.8%). Predation provided the lowest source of egg mortality (7.1%).
- Parasitism increased within seasons and between years. Sex ratio of all parasitoids increased in female bias from 2012 to 2013.
- *Anastatus* spp. accounted for 98.3% of parasitism (determined from enclosed parasitoids) suggesting that these native eupelmids contribute to biological control of *H. halys* in nurseries.

## Introduction

Invasive species have been well documented for their detrimental impacts in natural and managed ecosystems and for economic consequences associated with their introductions (Pimentel et al. 2005; Aukema et al. 2011). The successful invasion of exotic species into new regions is often attributed, partly, to their escape from natural enemies in their native range where they have a shared evolutionary history (Keane and Crawley 2002). Consequently, re-establishing population control in the invaded range can be an important tool for managing invasive species and providing significant economic benefits (Naranjo et al. 2014).

The brown marmorated stink bug, *Halyomorpha halys* (Stål), (Hemiptera: Pentatomidae), is an invasive species first detected in the USA in 1996 in Allentown, PA (Hoebeke and Carter 2003; Hamilton 2009). *Halyomorpha halys* is a highly polyphagous, utilizing over 100 host plant species or cultivars across its native and introduced ranges (Bergmann et al. 2013; Rice et al. 2014; Haye et al. 2015; Bergmann et al. 2016) and economically damaging pest (Leskey et al. 2012; Rice et al. 2014; Haye et al. 2015). Host plants include fruit, row and vegetable crops, and ornamental plants in natural and managed environments (Leskey et al. 2012; Rice et al. 2014; Bergmann et al. 2016). *Halyomorpha halys* use over 88 woody ornamental plants as feeding and/or oviposition hosts throughout the season (Bernon 2004; Bergmann et al. 2016).

In its native range of Japan, China, and Korea (Hoebeke and Carter 2003), *H. halys* has several natural enemies, particularly scelionid egg parasitoids such as *Trissolcus* spp. (Hymenoptera: Scelionidae). Studies performed within its native range show that egg parasitoids may be effective natural enemies of *H. halys* with parasitism rates as high as 84.7% (Zhang et al. 1993; Chu et al. 1997; Qiu 2007; Hou et al. 2009), suggesting biological control has a potential role in invaded ranges to keep *H. halys* in check. Classical biological control, which entails releasing natural enemies from the home land of the introduced pest, often takes multiple years of host specificity testing and other risk assessment procedures before initial releases of exotic natural enemies can begin (Kenis et al. 2017). Efforts toward classical biological control are well underway in the USA (Rice et al. 2014; Talamas et al. 2015, Herlihy et al. 2016; Santacruz et al. 2017). Recently, an adventive population of *Tr. japonicus*, a parasitoid of *H. halys* in its native range, has been confirmed in several US states (Talamas et al. 2015; Herlihy et al. 2016; Milnes and Beers 2016). The distribution, host use, and impact of this adventive population of *Tr*. *japonicus* are currently under study (P. Shrewsbury, unpublished).

Though classical biological control is an option, it is also important to elucidate native natural enemies impacts on *H. halys* within its invaded range, and ultimately their potential use in augmentative (rearing and release of native parasitoids) or conservation (habitat manipulation to favor parasitoids) biological control approaches. Several studies have been conducted to examine the identity and impact of native natural enemies on *H. halys* survival in various habitats (Jones et al. 2014; Cornelius et al. 2016; Herlihy et al. 2016; Morrison et al. 2016; Ogburn et al. 2016) within the USA. Overall, impacts on *H. halys* egg mortality tend to vary among habitat types, between predators and parasitoids, and by parasitoid species (Jones et al. 2014; Cornelius et al. 2016; Herlihy et al. 2016; Ogburn et al. 2016). Some of this variation may in part be explained by the diverse types of *H. halys* egg mass used in the studies. For example, when live sentinel (laboratory reared) was compared to naturally laid egg masses of *H. halys* in the field, it was found that parasitism rates, parasitoid community composition, and parasitoid species richness were significantly less for sentinel compared with naturally laid egg masses (Jones et al. 2014).

Ornamental plants play an essential role in the biology and ecology of *H. halys*. As such, it is important to understand the population dynamics of native natural enemies in ornamental plant nurseries and their potential for use in biological control programs. However, to our knowledge, only one previous study has been conducted to examine natural enemy activity in woody ornamental production nurseries (Jones et al. 2014). Nurseries often consist of diverse tree species offering the ability to examine patterns of natural enemy activity on different host plants within a common environment. The present study was conducted to: (1) evaluate mortality factors (e.g., predation, parasitism) affecting *H. halys* eggs, (2) identify indigenous parasitoids associated with *H. halys* eggs, and (3) quantify the impact of egg parasitoids on *H. halys* egg mortality in ornamental nurseries. Effects of study site location and host tree species on egg mortality and how the exposure period of eggs to predation and parasitism impact their mortality was also examined.

## Materials and methods

### Study sites and experimental design

Three field sites in Maryland, USA, were selected for this study. Selected nurseries were known to contain high populations of *H. halys* and an associated high abundance of naturally laid *H. halys* egg masses, which allows for a more realistic and statistically rigorous estimate of parasitoid activity (Jones et al. 2014; Herlihy et al. 2016). The nursery sites and tree species sampled were the same as those used in Jones et al. (2014) which provides a detailed description of sites and trees. Nursery sites included Raemelton Farm in Adamstown, MD (Frederick County) and two additional sites at Ruppert Nurseries in Laytonsville, MD (Montgomery County). Three genera of trees were chosen for monitoring at each nursery site based on preliminary observations that *H. halys* used these genera as oviposition hosts (Shrewsbury, unpublished). Trees included *Acer rubrum* L. ‘Franksred’ (red maple), *Prunus serrulata* L. ‘Kwanzan’ (ornamental cherry), *Ulmus americana* L. ‘Princeton’ (American elm), and *Ulmus parvifolia* Jacq. ‘Patriot’ (Chinese elm). Two species of elms were used as there were a limited number of any one species at the different sites. The number of trees surveyed for each species varied between nurseries due to differences in abundance of test species at each nursery (Table 1). Each field site was sampled approximately twice weekly throughout the 2012 and 2013 season (from late May to early September) which coincides with the period when *H. halys* is active (Leskey et al. 2015; Bergh et al. 2017).

**Table 1:** Total number of trees sampled at each of the three nursery sites by species

	Tree species				
Site	*Acer rubrum*	*Prunus serrulata*	*Ulmus americana*	*Ulmus parvifolia*	Total
1	19	47	73	–	139
2	28	20	–	31	79
3	87	70	100	–	257
Total	134	137	173	31	475

Site 1 = Raemelton Farm, Site 2 = Ruppert Nursery A, and Site 3 = Ruppert Nursery B

### Field sampling of trees for *H. halys* eggs

At each sampling event, all tree foliage up to a height of 2.7 m was searched thoroughly for egg masses laid in the field by *H. halys*. A small percentage of the naturally laid egg masses (92 of 2105 = 4.4%) assessed in this study were used to make comparisons to sentinel egg masses in Jones et al. (2014). At each sampling period, all egg masses present on a tree were assessed. The number of egg masses on a tree varied over time and overall ranged from 0 to 5 egg masses per tree. When located, the leaf containing the egg mass was marked with a piece of colored tape, and the location of the egg mass within the tree was recorded. Egg masses were left in the field for one of two exposure times: either 48 h (post-marking) or until *H. halys* hatched from the egg mass (7 days post-marking). These time periods were chosen to fully assess types of mortality that could vary temporally (48-h time period reducing the potential loss of parasitized eggs to predator consumption, and eggs left out until *H. halys* hatched were targeting mortality potentially due to predation. Egg masses were then collected from trees and transported to the laboratory for further assessment.

### Assessment of egg fate

In the laboratory, each egg mass was placed in a Petri dish (Thermo Fisher Scientific Incorporated, 100 mm × 15 mm) in a Percival incubator (25 °C ± 2 °C, 16L: 8D). Individual eggs in each mass were monitored to determine their fate. Emerging *H. halys* nymphs and parasitoids were removed and unhatched eggs were assessed for other causes of mortality. Parasitoids that emerged were placed in 70% ethanol for later identification. Egg parasitoids were identified according to Dieckhoff (unpublished). The number of parasitoids emerging and their sex were recorded. Females were identified to species, and males were pooled by genus due to difficulty in identification and the lack of taxonomic keys for males. Sex ratio was determined at the genus level since males could be accurately identified to genus but not to species. Eggs where nothing emerged or hatched were further assessed to determine other potential causes of mortality.

**Fig. 1:**
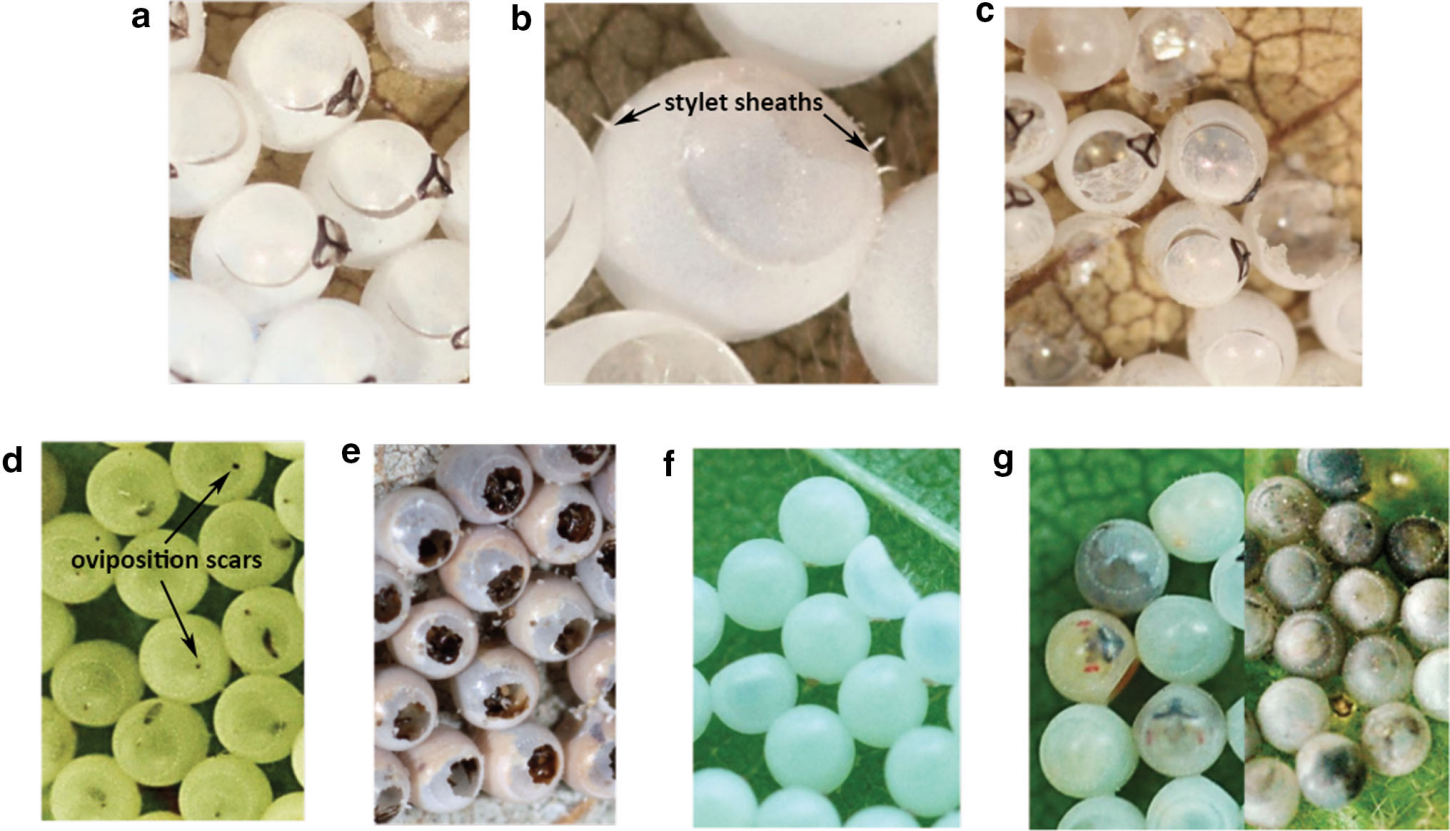
Examples of brown marmorated stink bug (*Halyomorpha halys*) egg mortality factors. Shown are: **a** eggs where nymphs emerged (no mortality), **b** predation by haustellate insects with stylet sheaths protruding from eggs, c predation by mandibulate insects with jagged holes present on eggs, **d** parasitized eggs with brown oviposition scars and discoloration, **e** eggs with parasitoid emergence (exit holes have been chewed from discolored eggs), **f** indented eggs where mortality is unknown, and **g** unascribed mortality (eggs with varying discoloration and no signs of predation or parasitism)

Egg fate and mortality factors were assigned to individual eggs within each egg mass with the use of a stereo microscope. Characteristics used in this study concur with those depicted by Morrison et al. (2016) for signs of predation by chewing and sucking predators. Unlike Morrison et al. (2016) this study also characterizes signs of parasitism. Egg fate categories included: “hatched” where *H. halys* emerged from the egg with no apparent mortality (Fig. 1a), “sucked” where there were three or more stylet sheaths protruding from the egg indicating a sucking predator had attacked the egg (Fig. 1b), “chewed” where eggs had been clearly fed upon by an organism with chewing mouthparts (Fig. 1c), “parasitized” where there was a distinct dark colored oviposition scar protruding slightly from the egg (Fig. 1d) or parasitoid emergence had occurred (Fig. 1e), and “unascribed” where eggs were unhatched and a direct cause of mortality could not properly be diagnosed. If there was no emergence of *H. halys* or parasitoids, eggs were dissected for signs of parasitism. If signs of parasitism were present eggs were classified as “parasitized” and if possible the “egg content” was identified to genus. If there were no signs of parasitism, they were considered “unascribed.”

### Statistical analysis

Percent egg mortality and parasitism per week in 2012 and 2013 were analyzed using linear regression. Each sampling “week” was defined as all sampling conducted within a period of 7 days and consisted of up to two visits to each site. We compared mortality among different fate categories (parasitized, chewed, sucked, and unascribed) by year, tree species, nursery site, and egg exposure time using *χ* ^2^ tests. Additionally, we tested the effects of year, tree species, site, and egg exposure time on each fate category using generalized linear models with a quasibinomial error distribution (Crawley 2012). Significance was then assessed with likelihood ratio *χ* ^2^ and type II sums of squares. Tukey HSD tests were conducted when significant effects were observed. All analyses were conducted using R 3.3.2 (R Core Team 2016).

## Results

**Fig. 2:**
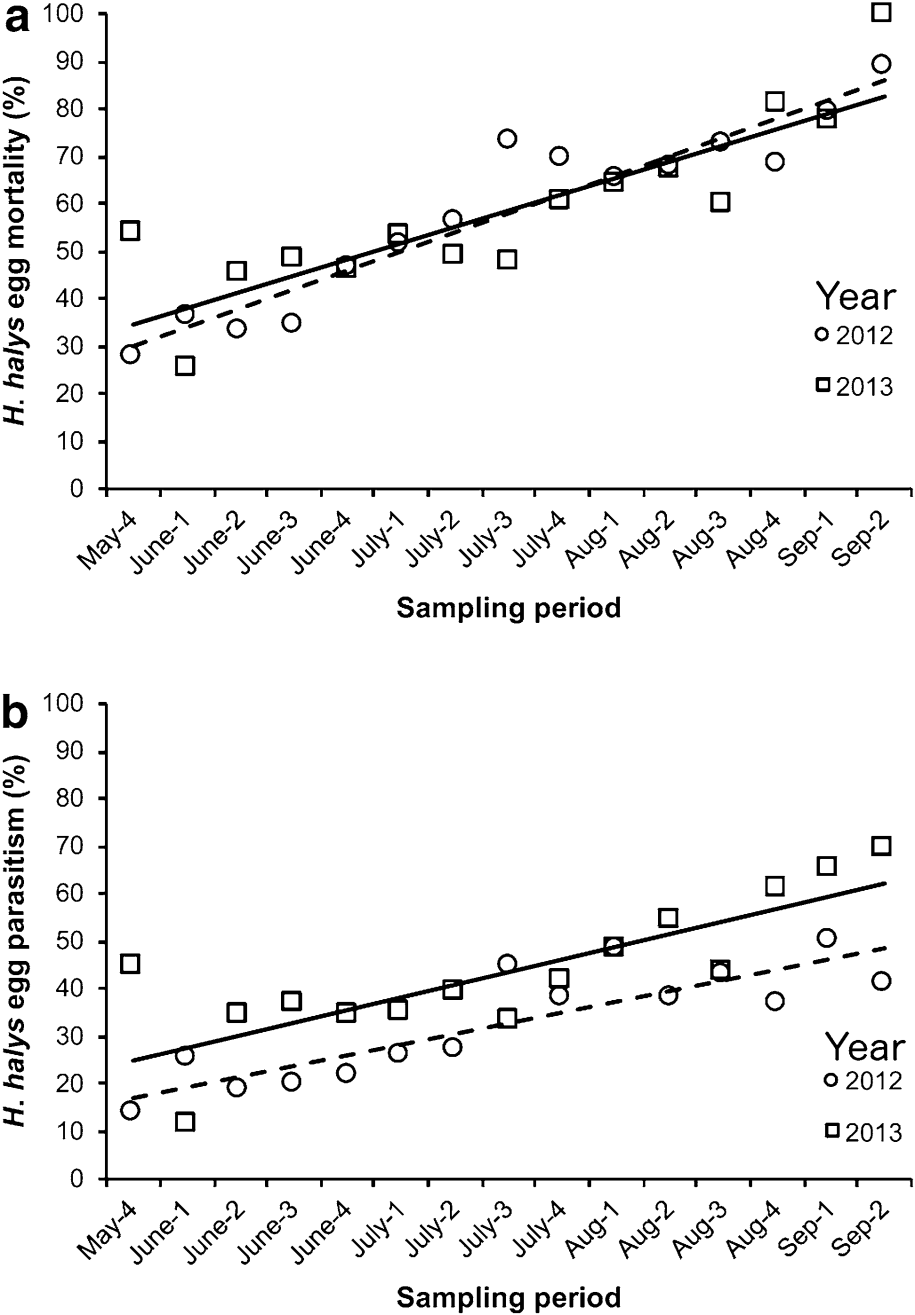
Shown are: **a** percent mortality of brown marmorated stink bug (*Halyomorpha halys*) eggs over time in 2012 (*y* = 4.00x + 25.99, *R* ^2^ = 0.892, *df* = 1, *p* < 0.001) and 2013 (*y* = 3.43x + 31.14, *R* ^2^ = 0.709, *df* = 1, *p* < 0.001), and **b** percent parasitism of brown marmorated stink bug eggs over time in 2012 (*y* = 2.25x + 14.74, *R* ^2^ = 0.718, *df* = 1, *p* < 0.001) and 2013 (*y* = 2.66x + 22.19, *R* ^2^ = 0.628, *df* = 1, *p* < 0.001). The number following each month indicates the week of that month in which sampling occurred, i.e., May-4 is the fourth week in May

A total of 897 and 1208 *H*. *halys* egg masses (24,124 and 32,073 eggs) were collected in 2012 and 2013, respectively (a total of 2105 egg masses and 56,197 eggs over 2 years). In both years, percent egg mortality was positively associated with sampling period (Fig. 2a). Percent egg mortality from all factors ranged from 27.93 to 88.89% per sampling period in 2012 and 25.03–100% per sampling period in 2013 (Supplementary Table 1), while the overall mean percent egg mortality for each year was 53.83 and 54.44% in 2102 and 2013, respectively.

**Fig. 3:**
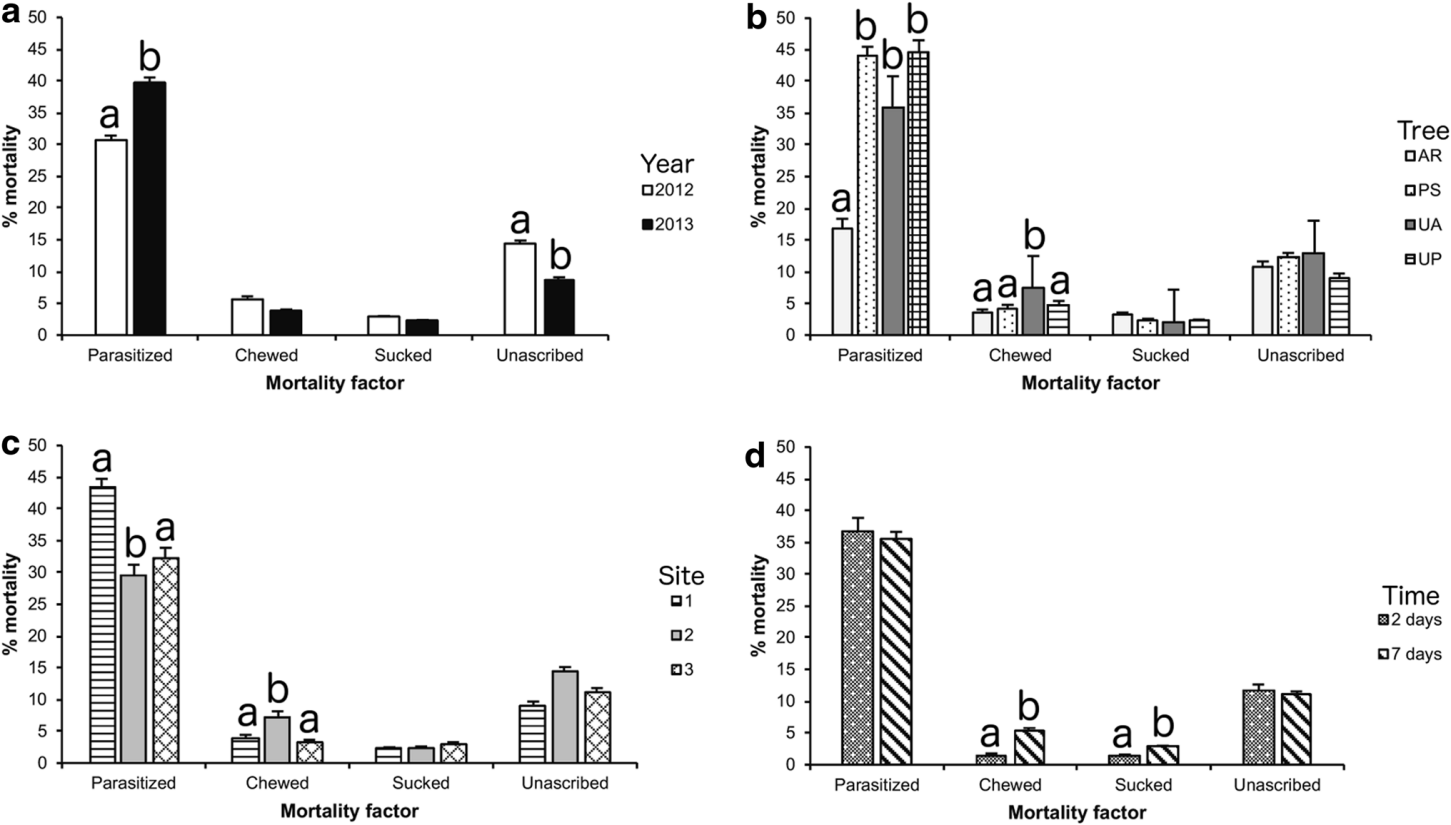
Fates of brown marmorated stink bug (*Halyomorpha halys*) eggs by: **a** year, **b** tree species, **c** site, and **d** egg exposure time. Mean percent egg mortality for each category of mortality pooled by week. *Bars represent* means and *lines* represent standard errors. For each mortality factor separately, different *lowercase letters* represent factors that were significantly different from each other (*p* < 0.05). Tree species are: *AR Acer rubrum*, *PS Prunus serrulata*, *UA Ulmus americana*, and *UP Ulmus parvifolia*. Sites are: 1 = Raemelton Farm, 2 = Ruppert Nursery A, and 3 = Ruppert Nursery B

There were significant differences among *H. halys* egg fates for mortality factors by year (*χ* ^2^ = 13,565.00, *df* = 3, *p* < 0.001; Fig. 3a), tree species (*χ* ^2^ = 1267.90, *df* = 9, *p* < 0.001; Fig. 3b), site (*χ* ^2^ = 2365.20, *df* = 6, *p* < 0.001; Fig. 3c), and egg exposure time (*χ* ^2^ = 363.76, *df* = 3, *p* < 0.001; Fig. 3d). In 2012 and 2013 parasitism was by far the greatest source of mortality for *H. halys* eggs (mean = 35.84%) followed by unascribed mortality (mean = 11.20%), and predation from chewing (mean = 4.61%) and sucking (mean = 2.53%) predators (Fig. 3a).

Parasitism was higher in 2013 than 2012 (*χ* ^2^ = 34.16, *df* = 1, *p* < 0.001; Fig. 3a), and was also higher on *P*. *serrulata*, *U*. *americana*, and *U*. *parvifolia* compared with *A*. *rubrum* host trees (*χ* ^2^ = 195.81, *df* = 3, *p* < 0.001; Fig. 3b). Parasitism was also higher at site 1 (Raemelton Farm) and site 3 (Ruppert Nursery B) compared with site 2 (Ruppert Nursery A) (*χ* ^2^ = 55.14, *df* = 2, *p* < 0.001; Fig. 3c). Egg exposure time did not affect parasitism (*χ* ^2^ = 0.58, *df* = 1, *p* = 0.446; Fig. 3d).

Although predation by chewing predators was relatively low, it was higher on *U. americana* compared with the other host trees (*χ* ^2^ = 8.08, *df* = 3, *p* = 0.044; Fig. 3b) and was also higher at site 2 (Ruppert Nursery A) than the other 2 sites (*χ* ^2^ = 25.90, *df* = 2, *p* < 0.001; Fig. 3c). We also found that chewing predation was greater when eggs were left exposed for 7 days compared with 2 days (*χ* ^2^ = 26.79, *df* = 1, *p* < 0.001; Fig. 3d). There was no effect of year on chewing predation (*χ* ^2^ = 2.02, *df* = 1, *p* = 0.156; Fig. 3a).

Sucking predation was only significantly affected by egg exposure time (*χ* ^2^ = 12.80, *df* = 1, *p* < 0.001; Fig. 3d), with more sucking predation when eggs were exposed to natural enemies for 7 days (2.80%) compared with 2 days (1.40%). Year (*χ* ^2^ = 0.03, *df* = 1, *p* = 0.854; Fig. 3a), tree species (*χ* ^2^ = 2.24, *df* = 3, *p* = 0.524; Fig. 3b), and site (*χ* ^2^ = 2.11, *df* = 2, *p* = 0.349; Fig. 3c) did not significantly affect sucking predation.

Unascribed mortality was only affected by year (*χ* ^2^ = 28.63, *df* = 1, *p* < 0.001; Fig. 3a), with 14.50% in 2012 compared with 8.75% in 2013. Tree species (*χ* ^2^ = 5.48, *df* = 2, *p* = 0.140; Fig. 3b), site (*χ* ^2^ = 4.93, *df* = 2, *p* = 0.085; Fig. 3c), and egg exposure time (*χ* ^2^ = 0.09, *df* = 1, *p* = 0.760; Fig. 3d) did not significantly affect unascribed mortality.

As parasitism represented the largest source of mortality, we examined how it varied by sampling period each year. In both years, percent parasitism increased over time (Fig. 2b). Percent egg parasitism ranged from 13.89 to 50.21% in 2012 and 11.31–69.37% in 2013. Parasitism of egg masses (one or more eggs parasitized within the egg mass) was 47.05% in 2012 and 52.48% in 2013. We observed 15,357 enclosed parasitoid adults from egg masses in this study (5638 and 9719 in 2012 and 2013, respectively). The species observed included seven native parasitoids: *A. reduvii*, *A. pearsalli, A. mirabilis, Tr. brochymenae, Tr. euschisti, Te. podisi,* and *Ooencyrtus* sp. (Table 2). The most abundant species by far was *A. reduvii* comprising 61.17 and 79.12% of all parasitoids in 2012 and 2013, respectively (Table 2). *Anastatus* spp. account for 98.32% of parasitism as determined from eclosed parasitoids. The sex ratio of the most dominant genus of parasitoid, *Anastatus* spp. (male: female) was 1:2.05 in 2012 and 1:4.86 in 2013. Although the other genera of parasitoids were far less abundant than *Anastatus*, a similar trend was also observed for *Telenomus* spp. (1:1 in 2012, and 1:10.5 in 2013) and *Trissolcus* spp. (1:9.44 in 2012, and 1:29.75 in 2013).

**Table 2:** Summary of the abundance and identity of eclosed hymenopteran parasitoid adults collected from brown marmorated stink bug (*Halyomorpha halys*) eggs in ornamental nurseries in Maryland

		2012		2013	
Genus	Species	*n*	Relative abundance (%)	*n*	Relative abundance (%)
*Anastatus*	*mirabilis*	46	0.82	119	0.63
	*pearsalli*	218	3.87	215	1.98
	*Reduvii*	3449	61.17	9031	79.12
	sp.	1811	32.12	208	16.77
*Ooencyrtus*	sp.	16	0.28	0	0
*Telenomus*	*podisi*	2	0.04	21	0.19
	sp.	2	0.04	2	0.05
*Trissolcus*	*brochymenae*	64	1.14	103	0.96
	*euschisti*	21	0.37	16	0.17
	sp.	9	0.16	4	0.14
Total		5638		9719	

## Discussion

Biological control is considered to be a sustainable or long-term solution for managing local and invasive pest species. Classical biological control is most commonly thought of for the mitigation of exotic pests (Kenis et al. 2017). However, some may contend it is equally important to consider augmentative or conservation biological control tactics to suppress invasive pest species. Toward this end, it is essential to identify and determine the impact of native natural enemies associated with a pest in its invaded range. This will provide insight on the likelihood that one or more native natural enemies can be successful used in augmentative or conservation biological control programs.

This study provides one of the most rigorous assessments of the identity and impact of indigenous natural enemies on *H*. *halys* egg mortality. More than 56,000 naturally laid eggs were monitored in woody ornamental nurseries. *Halyomorpha halys* egg mortality overall averaged 54% both years of the study and parasitism was markedly the greatest cause of egg mortality (35.8%), compared to chewing and sucking predation (7%) or unknown causes (11.2%). In both years, parasitism increased over the season and from 2012 to 2013. In addition to seasonal differences, parasitism rates were influenced by tree species, study site location, and egg exposure time in the field. Although seven native parasitoid species emerged from *H. halys* eggs during the 2-year study (Table 2), *A. reduvii* was the most abundant, making up 61.2 and 79.2% in 2012 and 2013, respectively. The 3 *Anastatus* spp. as a group accounted for 98.3% of parasitism.

Results from the present study are similar to an earlier, but much smaller, study in ornamental plant nurseries that compared the parasitoid community and parasitism rates on naturally field laid egg masses to sentinel (live) egg masses of *H. halys* (Jones et al. 2014). In the latter study, naturally laid eggs of *H. halys* incurred parasitism rates as high as 28.4 and 55.3% over the 2-year study and six native egg parasitoids were detected parasitizing *H. halys* eggs. *Anastatus* spp. made up 99.3% of the parasitoid community, while scelionid egg parasitoids rarely (0.7%) emerged from *H. halys* eggs. As with the present study, A. *reduvii* was also the most abundant parasitoid species (Jones et al. 2014).

Other studies have been conducted to examine the community composition of parasitoids and other native natural enemies associated with *H. halys* in the mid-Atlantic and north east region of the U.S. Studies have been conducted in a range of habitat types (woody plots, landscape plots, and fruit, vegetable, and row crop systems) (Cornelius et al. 2016; Herlihy et al. 2016; Ogburn et al. 2016). Although a variety of factors may affect species of parasitoids found within a habitat, there is data suggesting that some of these species are habitat specialists, and *H*. *halys* egg parasitism has generally been found to be higher in arboreal habitats (Cornelius et al. 2016; Herlihy et al. 2016). For example, in the present study in nurseries which are arboreal habitats, *Anastatus* spp. followed by *Trissolcus* spp. were the most abundant, while other genera (*Telenomus* and *Ooencyrtus*) were found at very low abundances (0–0.28% of parasitoids). Similarly, research in two other types of arboreal habitats, experimental landscape plots and woodlots, found just two parasitoid species, *Tr. euschisti* and *A. reduvii*, in both habitats (Cornelius et al. 2016). Additionally, Herlihy et al. (2016) studied parasitoid activity on *H. halys* eggs in three habitat types, woods, orchards (both arboreal), and soybean fields, and found 2 native (*Tr. euschisti* and *Tr. brochmymenae*) and 1 exotic (*Tr. japonicus*) parasitoid occurred more often in woods habitat, *A. reduvii* was exclusively in orchard, and *Te. podisi* was exclusively recovered in soybean. Therefore, these studies including the present depict a preference of *Anastatus* and *Trissolcus* species for arboreal habitats, and *Telenomus* and *Ooencyrtus* species for agricultural crops which support of earlier findings (Okuda and Yeargan 1998; Serrano and Foltz 2003; Qiu 2007; Hou et al. 2009; Maltese et al. 2012; Danne et al. 2013).

Within a habitat, host plant composition can influence rates of parasitism and predation. We found significant differences in parasitism among different host trees with *Acer* having the lowest rates and *Prunus* and *Ulmus* having significantly higher rates (Fig. 3b). Our results support variation noted by Cornelius et al. (2016) who also found parasitism was significantly higher on *Prunus* compared to *Acer.* This difference was attributed mainly to *A. reduvii* which was the most prevalent native parasitoid recovered (Cornelius et al. 2016). These results suggest other factors besides an arboreal habitat, may also be influencing the activity of *A. reduvii* such as differences in *H. halys* abundance on different tree species (Bergmann et al. 2016). Greater plant species richness has been shown to support greater abundance of natural enemies due largely to the presence of diverse communities of alternate prey (Shrewsbury and Raupp 2006). Ornamental plant nurseries provide a wide variety of host plants that support a diversity of Hemiptera and Lepidoptera that may serve as reservoirs of alternate hosts for *A. reduvii* and enhance its ability to impact *H. halys* in both nurseries and other arboreal habitats. Altogether, these results suggest that conservation biological control may be implemented to favor natural enemies such as incorporating tree species that support high rates of parasitism or alternate hosts for generalist natural enemies into the managed habitat.

In addition to habitat type, the type of egg mass used may also affect egg mortality. The present study used only egg masses that were laid naturally in the field, whereas other studies frequently used a combination of naturally laid and 2 types of sentinel egg masses [i.e., laboratory-reared eggs that were fresh (live) or frozen (dead)]. Some work has suggested that sentinel (live) egg masses of *H. halys* significantly underestimate parasitism rates, parasitoid community composition, and parasitoid species richness (Jones et al. 2014), while other research has found that native parasitoids emerge more frequently from frozen than fresh eggs (Haye et al. 2015; Herlihy et al. 2016).

There are several possible factors influencing the success rate of native parasitoids attacking exotic *H. halys* eggs in its invaded range. In the present study, *Anastatus* species, especially *A. reduvii*, were significantly more successful at completing development in *H. halys* eggs than *Trissolcus* species. A similar pattern was found in Europe where the only native parasitoids to successfully develop in *H. halys* were *A. bifasciatus* and *Ooencyrtus telenomicida* (Haye et al. 2015; Rondoni et al. 2017). *Anastatus reduvii* and *A. bifasciatus* are generalist egg parasitoids across several orders of insects (ex. Hemiptera, Lepidoptera, Orthoptera) (Howard 1880; Krombein et al. 1979; Mendel et al. 1989; Hou et al. 2009; Haye et al. 2015) whereas *Trissolcus* species are egg parasitoids specific to the superfamily Pentatomoidea (Yang et al. 2009). A possible explanation for differences in parasitism between native parasitoid species on exotic hosts is that generalist and specialist parasitoids differ in their ability to overcome or adapt to new host defensive responses (Vinson 1990; Haye et al. 2015). Generalist parasitoids interact with a variety of defensive responses from their hosts and should be better adapted at overcoming host defenses than specialist parasitoids that encounter fewer defenses (Vinson 1990; Haye et al. 2015). Not surprisingly, over time introduced species tend to be attacked by increasing numbers of native parasitoids as they likely adapt to defenses (Cornell and Hawkins 1993; Hawkins and Cornell 1994; Haye et al. 2015). This supposition is further supported by studies that show that native European and North American *Trissolcus* and other parasitoid species that do not successfully develop in naturally laid or sentinel live eggs are successful on frozen eggs whose defensive responses are likely compromised (Haye et al. 2015; Herhily et al. 2016). In contrast, the Asian *Tr. japonicus* and *Tr. cultratus* share a coevolved history with *H. halys* in Asia and have likely adapted to defensive responses of *H. halys*.

*Anastatus* species have a history of successful biological control as different species of *Anastatus* were released against several pest species with parasitism ranging from 23% to greater than 90% (Huang et al. 1974; Fay and De Faveri 1997; Qiu 2007; Hou et al. 2009; Danne et al. 2013). A native Asian *Anastatus* species was tested against *H. halys* in Beijing with parasitism rates ranging from 52.6 to 64.7% (Hou et al. 2009). In Europe, studies have shown the only native parasitoid to successfully develop consistently in *H. halys* is *A. bifasciatus* (Haye et al. 2015). In the present study, we observed *Anasatus spp.* parasitizing *H. halys* eggs at a rate of approximately 98%. Together these studies show strong support for *Anastatus* spp. as a good candidate for use in augmentative biological control programs targeting *H. halys*.

*Anastatus* and all other genera of parasitoids in our study showed an interesting change in sex ratios. This shift in sex ratio from male to female bias may be due to an increase in host resource (more *H. halys* eggs). Many ovipositing parasitoids respond to the traces of other females by increasing the proportion of male progeny (Hamilton 1967; Liljesthrom et al. 2013). Therefore, it is possible that male production decreased due to greater resource availability likely reducing the chances of female parasitoid interaction.

Findings from the present study suggest a number of interesting directions for future research. For instance, although interspecific interactions among parasitoids were not directly examined, parasitism rates or outcomes may be influenced by factors such as multi-parasitism, egg guarding by adult parasitoids, or competition between adult parasitoids (Abram et al. 2014; Haye et al. 2015; Konopka et al. 2016). Consequently, it will be especially interesting to study interactions between native parasitoids and the exotic *Tr. japonicas* that has adventive populations in the U.S. (Talamas et al. 2015; Herlihy et al. 2016; Milnes and Beers 2016). Additionally, in the present study, all unhatched eggs in which no parasitoids emerged, were dissected for signs of underdeveloped or developed parasitoids that may have killed the egg but failed to egress. If no parasitoid was found, the egg was classified as unascribed. As such, some of the unascribed mortality may have been caused by parasitoids. This conjecture is further strengthened by the fact that there was a change (increase) in parasitism rate from 2012 to 2013, which coincided with an inverse change (decrease) in unascribed egg mortality. If this was indeed the case it suggests no change in parasitism when direct and potential indirect effects of parasitoids are combined. Therefore, parasitism attempts by parasitoid species other than *Anastatus* may actually be more common than indicated by parasitoid emergence data (Abrams et al. 2014; Haye et al. 2015).

In summary, we quantified *H*. *halys* egg mortality and identified indigenous egg parasitoids attacking *H. halys* eggs in ornamental nurseries. The results of this study and others indicate that *Anastatus* spp., particularly *A. reduvii*, are strong candidates for use in biological control programs. *Anastatus* spp. are generalist parasitoids that provide high rates of parasitism of *H. halys* in nurseries, and have been used successfully in augmentative biological control programs against other Heteropteran pests in different systems. This is strong evidence that *A. reduvii* is an excellent candidate for use in augmentative biological control. Conservation biological control targeting not only *Anastatus* species but also predators may be possible in nursery or other arboreal habitats. *Anastatus* is arboreal, and parasitism rates were affected by habitat factors such as tree species (significantly lower on maple than other than other trees tested), and study location. Although predation rates were lower than parasitism, they still impacted *H. halys* egg mortality. Predation also was affected by habitat factors. These results suggest habitats could be manipulated to favor parasitoids and predators. Results from this study provide evidence that future research should focus on native *Anastatus* spp., particularly *A. reduvii*, and their use in both augmentative and conservation biological control programs, in addition to conservation of predators. The data on indigenous natural enemy communities attacking *H*. *halys* in the present study could provide a useful baseline for comparisons in studies on the interactions between the native *A. reduvii* and the exotic *Tr. japonicus* egg parasitoids and their impact on biological control of *H. halys* in the USA.

## Author contributions statement

All authors conceived and designed this study. ALJ conducted experiments. ALJ and DEJ analyzed the data. All the authors contributed substantially to the writing and editing of the manuscript. All authors approved the final manuscript

## Electronic supplementary material

Below is the link to the electronic supplementary material.

Supplementary material 1 (DOCX 19 kb)
