# Supplementary material for: Field surveys of egg mortality and indigenous egg parasitoids of the brown marmorated stink bug, Halyomorpha halys, in ornamental nurseries in the mid-Atlantic region of the USA
Source: J Pest Sci (2004). 2017 Jun 5;90(4):1159–68. doi: 10.1007/s10340-017-0890-8 (PMC5544785; doi:10.1007/s10340-017-0890-8)
Supplement: Supplementary file 1 — Supplementary material 1 (DOCX 19 kb) [file 10340_2017_890_MOESM1_ESM.docx]

Supplementary Table 1: Means (± SE) of *H*. *halys* mortality factors (%) per sampling period for 2012-2013.

| Sampling Period | Mortality factors | | | | | | | |
| --- | --- | --- | --- | --- | --- | --- | --- | --- |
|  | Parasitized | | Chewed | | Sucked | | Unascribed | |
|  | 2012 | 2013 | 2012 | 2013 | 2012 | 2013 | 2012 | 2013 |
| May-4 | 13.89 ± 9.59 | 44.35 ± 14.19 | 0 ± 0 | 4.17 ± 2.84 | 0 ± 0 | 1.38 ± 0.83 | 14.04 ± 7.74 | 3.65 ± 2.02 |
| June-1 | 25.05 ± 9.83 | 11.31 ± 6.32 | 0 ± 0 | 0 ± 0 | 0 ± 0 | 3.33 ± 0.99 | 11.10 ± 3.66 | 10.40 ± 3.85 |
| June-2 | 18.39 ± 2.65 | 34.60 ± 5.02 | 0.91 ± 0.44 | 0.75 ± 0.65 | 3.30 ± 0.54 | 2.01 ± 0.58 | 10.52 ± 1.37 | 8.17 ± 2.20 |
| June-3 | 20.02 ± 3.78 | 37.05 ± 4.98 | 0.46 ± 0.33 | 1.80 ± 1.04 | 4.19 ± 0.83 | 1.73 ± 0.43 | 9.78 ± 1.74 | 7.98 ± 2.07 |
| June-4 | 21.51 ± 4.21 | 34.44 ± 3.56 | 2.22 ± 1.03 | 1.33 ± 0.67 | 1.91 ± 0.58 | 2.38 ± 0.44 | 20.65 ± 3.08 | 7.79 ± 1.30 |
| July-1 | 25.64 ± 5.97 | 34.78 ± 3.94 | 2.81 ± 1.14 | 2.00 ± 0.67 | 2.52 ± 0.90 | 3.24 ± 0.68 | 20.33 ± 3.54 | 13.03 ± 2.00 |
| July-2 | 26.98 ± 3.02 | 39.39 ± 4.97 | 8.49 ± 1.74 | 2.36 ± 0.94 | 3.49 ± 0.71 | 1.74 ± 0.47 | 16.89 ± 1.71 | 5.33 ± 1.52 |
| July-3 | 44.44 ± 7.33 | 33.24 ± 3.16 | 8.84 ± 3.24 | 3.38 ± 0.94 | 3.29 ± 1.69 | 2.86 ± 0.55 | 16.57 ± 3.07 | 8.31 ± 1.32 |
| July-4 | 38.10 ± 3.97 | 41.74 ± 2.76 | 10.98 ± 2.40 | 5.00 ± 1.00 | 3.29 ± 1.21 | 2.58 ± 0.50 | 17.32 ± 1.74 | 11.20 ± 1.34 |
| August-1 | 48.48 ± 3.93 | 48.05 ± 3.62 | 3.05 ± 1.12 | 7.14 ± 1.47 | 2.27 ± 0.54 | 1.50 ± 0.41 | 11.32 ± 1.38 | 7.59 ± 1.19 |
| August-2 | 37.95 ± 8.28 | 54.48 ± 4.78 | 10.41 ± 5.06 | 5.53 ± 1.90 | 1.84 ± 1.01 | 1.02 ± 0.39 | 17.44 ± 4.58 | 5.89 ± 1.64 |
| August-3 | 42.66 ± 4.77 | 43.39 ± 8.41 | 10.10 ± 2.77 | 8.26 ± 3.97 | 2.80 ± 1.17 | 1.86 ± 0.99 | 16.75 ± 2.12 | 6.42 ± 2.03 |
| August-4 | 36.79 ± 6.89 | 60.84 ± 10.98 | 10.34 ± 3.73 | 8.65 ± 4.40 | 2.71 ± 1.78 | 5.13 ± 2.82 | 18.54 ± 3.70 | 6.47 ± 2.99 |
| September-1 | 50.21 ± 14.18 | 65.48 ± 12.64 | 15.90 ± 10.00 | 5.14 ± 5.14 | 0 ± 0 | 0 ± 0 | 13.26 ± 5.63 | 6.70 ± 2.44 |
| September-2 | 41.13 ± 9.14 | 69.37 ± 15.32 | 31.30 ± 8.58 | 15.48 ± 15.48 | 0 ± 0 | 0 ± 0 | 16.46 ± 5.24 | 15.15 ± 15.15 |
